# Predictors of Cognitive Resilience in the Multi-Ethnic Study of Atherosclerosis

**DOI:** 10.1093/geroni/igaf122.4288

**Published:** 2025-12-31

**Authors:** Leidys Gutierrez-Martinez, Maude Wagner, Hodan Mohamed, Timothy Hughes, Timothy Hohman, Rachel F Buckley, M Maria Glymour, Marcia Pescador Jimenez

**Affiliations:** Boston University, Boston, Massachusetts, United States; Rush University Medical Center, Chicago, Illinois, United States; Boston University, Boston, Massachusetts, United States; Wake Forest University School of Medicine, Winston-Salem, North Carolina, United States; Vanderbilt University Medical Center, Nashville, Tennessee, United States; Massachusetts General Hospital, Boston, Massachusetts, United States; Boston University, Boston, Massachusetts, United States; Boston University, Boston, Massachusetts, United States

## Abstract

Modifiable predictors of cognitive resilience (CR)—maintaining better-than-expected cognition despite neuropathology—may inform dementia prevention strategies. We quantified CR and identified main predictors in a U.S.-based multi-ethnic cohort. We used data from 1,335 individuals enrolled in 2000-02 (Exam-1; age=45-84) in the Multi-Ethnic Study of Atherosclerosis (MESA) with repeated global cognition scores (z-scores of Cognitive Abilities Screening Instrument, Digit Symbol Coding, Digit Span) administered at Exams 5 (2010-12), 6 (2016-18), and 7 (2022-24). Neuroimaging biomarkers assessed at Exam 6, included white matter fractional anisotropy, white matter hyperintensities, and total brain volume, adjusted for intracranial volume. First, using linear mixed-effects models adjusted for age, sex, and race/ethnicity, we estimated CR as random slopes of global cognition, capturing individual deviations from expected cognitive decline given a specific neuroimaging profile. CR estimates were z-scored; positive values indicated better CR. Second, using general linear models, we assessed whether modifiable social and health-related risk factors at Exam 1 predicted CR a decade later. Each predictor’s contribution to CR was assessed by percentage change in R-square by comparing fully-adjusted vs. reduced-models. At first neurocognitive assessment, study participants averaged 66 years old (SD = 8) and 46% were male. In fully-adjusted models, educational attainment (β = 0.19;95% CI=0.11,0.27) and family income (β = 0.12;95%CI=0.07,0.18) were independently associated with higher CR. More depressive symptoms and higher waist girth were independently associated with lower CR (β=-0.01;95% CI=-0.02,-0.01; β=-0.01;95% CI=-0.02,-0.01, respectively). Education and income were main contributors to CR (%R-squared Change=39% and 22%, respectively). Early favorable social (education, income) and health factors (depressive symptoms, waist girth) may promote CR decades later.

